# Genomic and secondary metabolites of the marine cyanobacterium *Capilliphycus salinus* ALCB114379

**DOI:** 10.1111/jpy.70174

**Published:** 2026-05-09

**Authors:** Gabriel Schimmelpfeng Passos, Rafael Barty Dextro, Mauricio Junior Machado, Anderson Miguel Teixeira Feitosa, Núbia Pereira Silva, Renata Beatriz Cruz, Ana Paula Dini Andreote, Ernani Pinto, Marli Fatima Fiore

**Affiliations:** ^1^ Center for Nuclear Energy in Agriculture (CENA) University of São Paulo (USP) Piracicaba SP Brazil

**Keywords:** microcyclamide, mycosporin, Oscillatoriales, phylogenomic, taxonomy

## Abstract

Assembling high‐quality genomes from underexplored environments can be helpful for understanding microbial diversity and identifying novel species. The Cyanobacterium type strain *Capilliphycus salinus* ALCB114379 is a representative of Oscillatoriales order isolated from a supralittoral zone of the south Atlantic Ocean in Brazil, an ecotone characterized by significant environmental fluctuations due to its transitional nature between the tidal and terrestrial environments. Here, we present its genome assembled into a single contig and circularized, with a total size of 7.7 Mb and 99.67% completeness. The genome has 6204 protein‐coding genes, including several involved in the biosynthesis of secondary metabolites. Despite being a homocytous genus, *C. salinus* ALCB114379 possesses a gene cluster for the biosynthesis of molybdenum‐type nitrogenase, indicating a potential for N_2_ fixation. Additionally, the genome contains other gene clusters related to the biosynthesis of biotechnologically relevant compounds, such as microcyclamide (*mca*), a cytotoxic cyanopeptide, and mycosporine‐like amino acids (MAAs), with photoprotective and antioxidant functions. High‐performance liquid chromatography–tandem mass spectrometry (HPLC–MS/MS) analysis showed the detectable production of three MAAs, with shinorine being the most abundantly produced compared with porphyra‐334 and palythine. This work provides insights into the Cyanobacteria phylogenomics and metabolism, highlighting the potential of *C. salinus* ALCB114379 as a source of specific metabolites for biotechnological applications.

**Abbreviations**
BGC: biosynthetic gene clusters
CCS: circular consensus sequencing
CDS: coding sequences
CENA: Center of Nuclear Energy in Agriculture
ESI: electrospray ionization source
GTDB: Genome Taxonomy Database
HGT: horizontal gene transfer
HPLC: high‐performance liquid chromatography
HPLC–MS/MS: high‐performance liquid chromatography–tandem mass spectrometry
LC: liquid chromatography
LOQ: limit of quantification
MAAs: mycosporine‐like amino acids
NCBI: National Center for Biotechnology Information
ORF: open reading frame
RefSeq: reference sequence
TQMS: triple quadrupole mass spectrometer

## INTRODUCTION

The successful description of cyanobacterial diversity focuses on exploring new habitats for sampling combined with the proper identification of isolated strains in culture collections. An example of this effort is the description of a new marine Cyanobacterial genus previously classified within the large *Lyngbya*‐like group (Berthold et al., 2022; Caires et al., 2018; Wang et al., 2023). The genus *Capilliphycus* resembles *Lyngbya*, but a polyphasic approach combining ecology, morphology, and genetics distinguished several species and grouped them based on shared traits such as the presence of hormogonia, sheaths around the cells, and high salt tolerance (Caires, 2019). *Capilliphycus salinus* ALCB114379 is the type strain of this genus (Caires et al., 2018; Caires, 2019). With further research, more species of *Capilliphycus* are being described, expanding its geographical distribution to salt flats and hypersaline ponds connected to the Atlantic Ocean, including locations in Brazil, the United States, and France (Berthold et al., 2022; Caires et al., 2018).

Sequencing type strains to produce high‐quality genome assemblies aids in the accurate taxonomic identification of Cyanobacteria. The genomic data of Cyanobacteria also serve as the basis for evolutionary inferences about ecological adaptations, including factors like light harvesting, resistance to salinity, and dehydration (Chen et al., 2021). Furthermore, the study of genomes can elucidate phenomena such as horizontal gene transfer (HGT), infectious processes, and organism co‐existence (Morimoto et al., 2020; Popin et al., 2021; Shoguchi et al., 2018). It is also relevant to note the scarcity of available genomes belonging to the Oscillatoriales order. Currently, there are only 26 genera of Cyanobacterial strains within this order with genome assemblies deposited on the National Center for Biotechnology Information (NCBI) platform (Release 263.0). This represents a constraint for research focused on diversity within this order, particularly those recognized as polyphyletic (Komárek, 2023).

Coastal marine environments, recognized as some of the most dynamic ecosystems on Earth, are home to a wide range of marine microorganisms that perform functions associated with energy transformation and the maintenance of biogeochemical cycles. Among these microorganisms, cyanobacteria are vital agents for sustaining ecosystems through nitrogen and carbon fixation (El‐Seedi et al., 2023; Thornton, 2014). Furthermore, their presence in diverse habitats worldwide is predominantly recognized for the genetic diversity they exhibit, combined with their ability to synthesize ecologically significant natural compounds (El‐Seedi et al., 2023; Tan & Phyo, 2020). An example of such metabolites is the mycosporine‐like amino acids (MAAs), a group of water‐soluble UV‐absorbing chromophores (Rosic, 2021). These antioxidant photoprotective compounds are essential for the successful colonization of habitats with high solar exposure, such as the intertidal coastal zones (Görünmek et al., 2024).

In this study, we sequenced and assembled the genome of the Cyanobacterium *Capilliphycus salinus* ALCB114379, the type strain for this genus. These data provide a comprehensive basis for taxonomic elucidation through phylogenomics and also allowed the prediction of biosynthetic gene clusters (BGCs) for the production of ecologically significant natural compounds, shedding light on the metabolic capabilities of this organism.

## MATERIALS AND METHODS

### Cyanobacterium cultivation

The *Capilliphycus salinus* ALCB114379 strain is maintained in the Cyanobacterial Culture Collection of the Center of Nuclear Energy in Agriculture (CENA) and corresponds to the reference strain described by Caires et al. (2018) and Caires (2019), originally isolated from hypersaline tidal pools in a supralittoral zone in Bahia State, Brazil. Cultures were maintained in Z8 medium (Kotai, 1972) under a 14:10 h light:dark cycle, fluorescent light (45 ± 5 μmol photons · m^−2^ · s^−1^), and 22 ± 1°C. Cells were grown in 125‐mL flasks containing 50 mL medium for 20 days and harvested by centrifugation.

### DNA extraction, genome sequencing, and assembly

After growth, the biomass was concentrated by centrifugation, and cell washing was performed according to Heck et al. (2016), with the intent to reduce the bacteria surrounding the surface of the trichomes. Total genomic DNA (gDNA) was extracted using the All‐Prep DNA/RNA Mini kit (Qiagen, Hilden, Germany) according to the manufacturer's instructions. The integrity of the extracted gDNA was evaluated using electrophoresis on a 1% (w/v) agarose gel and quantified with the Qubit® 2.0 Fluorometer (Life Technologies, Carlsbad, California, United States). To preserve gDNA integrity during transport, DNAstable Plus (Biomatrica, San Diego, California, United States) was applied. The gDNA was then sent for whole‐genome sequencing to the Joint Genome Institute, where a PacBio SMRTbell library was prepared for circular consensus sequencing (CCS) and sequenced using the PacBio HiFi platform. Post‐sequencing data analysis included quality filtering of reads with BBMap v38.90, employing the icecreamfinder.sh pipeline with default parameters (Liu et al., 2019).

Reads were taxonomically classified using Kaiju v1.2.9 with the reference sequence (RefSeq) database to evaluate possible non‐Cyanobacterial gDNA contamination, likely from Cyanobacteria‐associated bacteria that persisted after the washing procedure (Menzel et al., 2016). Due to the absence of reference sequences, de novo genome assembly was performed using HiFiASM v0.16.1 with default parameters (Cheng et al., 2021). To classify the assembled scaffolds and verify the integrity of the resulting sole circular chromosome, single‐copy orthologous genes of Cyanobacteria were identified via Benchmarking Universal Single‐Copy Orthologs (BUSCO v5.3.; Simão et al., 2015). The circularity of the assembled genome was confirmed using Bandage (Wick et al., 2015). Completeness and contamination levels were assessed with CheckM v1.0.18 (Parks et al., 2015). The quality of the assembly was evaluated through Quast v5.0.2 (Gurevich et al., 2013), providing information on genome size and GC content. To evaluate the coverage of the genome using the raw sequencing data, we first aligned the assembled genome to the raw sequencing reads with Minimap2 v2.28 (Li, 2018). Subsequently, Qualimap v2.3 was employed to calculate the coverage, providing a comprehensive assessment of the sequencing depth across the assembled genome (García‐Alcalde et al., 2012). The genome map with GC skew was generated using Proksee (Grant et al., 2023). Finally, the taxonomic placement of the assembled circular genome was determined using the bac120_markers file of the Genome Taxonomy Database (GTDB; Chaumeil et al., 2020).

### Phylogenetic characterization

To construct the phylogenetic tree based on the 16S rRNA gene, 130 sequences were selected from the National Center for Biotechnology Information (NCBI) and aligned using MUSCLE (Edgar, 2004) with the MEGA 7.0.26 software. The substitution model applied was Kimura‐2 Gamma distributed with Invariant sites (G + I). The phylogenetic analysis was performed using the maximum likelihood with a bootstrap of 1000. Bayesian inference was performed using MrBayes v3.2.7a software (Ronquist et al., 2012). The analysis included 500,000 replicates to estimate the robustness of the resulting branches. The evolutionary model applied was the General Time Reversible (GTR) model, with a gamma‐distributed rate variation among sites and a proportion of invariant sites (GTR + I + Γ). Two independent runs of the Markov chain Monte Carlo were conducted, each with four chains (one cold and three heated), sampling every 500 generations. To ensure convergence, the first 25% of trees were discarded as burn‐in. Posterior probabilities were calculated to assess the support for individual branches.

For the phylogenomic analysis, amino acid sequences of 120 bacterial single‐copy conserved markers were aligned using GTDB‐tk v0.3.2 (Chaumeil et al., 2020). These aligned protein sequences were then used to place *Capilliphycus salinus* ALCB114379 within a maximum likelihood phylogenomic tree. The tree was constructed using RAxML v8.0.0, with default parameters and 1000 bootstraps to ensure robust phylogenetic inference (Stamatakis, 2014). For this analysis, 97 Cyanobacterial genomes were selected from NCBI, each with more than 90% completeness and <6% contamination, to provide a comprehensive and reliable phylogenetic context. Both trees (16S rRNA gene and genome) were edited using ITOL v6.5.8 (Letunic & Bork, 2019).

### Genome annotation

The structural and functional annotation of the *Capilliphycus salinus* ALCB114379 genome was performed using Prokka v1.14.6 (Seemann, 2014). The predicted proteins were categorized into biological subsystems via BlastKOALA v2.2 (Kanehisa et al., 2016). Sequences of biotechnological relevance were manually curated using Artemis v18.2.0 (Carver et al., 2012). InterPro was used to identify conserved domains within the annotated clusters, and the selected sequences were subjected to a synteny analysis using Clinker v0.0.28. This analysis compared the sequences with their closest related counterparts available in the NCBI database (Gilchrist & Chooi, 2021). To identify potential HGT events, codon‐bias analysis was performed on the gene(s)/cluster of interest using Codon Usage (https://www.bioinformatics.org/sms2/codon_usage.html). In addition, a genome‐wide compositional screen for putative horizontally acquired regions was performed with Alien Hunter (Vernikos & Parkhill, 2006). Finally, an automatic untargeted BGC search was performed using antiSMASH in strict mode (Blin et al., 2021; Zhang et al., 2022).

### Mycosporine‐like amino acid extraction and analysis

Cyanobacteria cultivation was performed as described previously. Centrifugation was used to concentrate biomass, and the supernatant was discarded. Then, the obtained biomass was lyophilized. A total of 5 mg of the lyophilized biomass was weighed into 2‐mL polypropylene microcentrifuge tubes (*n* = 3). The extraction was performed with 2.0 mL of 0.1% (v/v) formic acid and 0.2 mM ammonium formate (pH ~2.55) homogenized by vortex. After 1 h at room temperature (~22 ± 1°C), the samples were mixed in a vortex for 30 s, disruption by one cycle of sonication at 30% amplitude for 3 min (Omni Sonic Ruptor 400, Omni, Kennesaw, Georgia, United States), and mechanically disrupted with two 5‐mm stainless steel beads in each microcentrifuge tube for 5 min with oscillation frequencies of 30 Hertz in TissueLyser LT system (Qiagen, Hilden, Germany). The extracts were centrifuged (at 10,000 rpm for 10 min at 4°C), and the supernatant was filtered to remove cell debris (0.45‐μm polyvinylidene fluoride filter). The final extract was spiked with 10 μL of internal standard solution (100 μg · mL^−1^ aciclovir). Quantitative analysis of three different MAAs (shinorine, porphyra‐334, and palythine) in the extracts (using standards for each compound) was performed using a 1290 series liquid chromatography (LC) system equipped with a 1290 VL pump and a 1260 high‐performance autosampler (HiP ALS) injector system coupled to a 6460 triple quadrupole mass spectrometer (TQMS; Agilent Technologies, Santa Clara, California, United States) with an electrospray ionization source (ESI) as described by Geraldes et al. (2020). Finally, Tukey's test was applied to assess whether there was a significant difference in production among the identified MAAs.

## RESULTS

### *Capilliphycus salinus* ALCB114379 assembled genome

**FIGURE 1:**
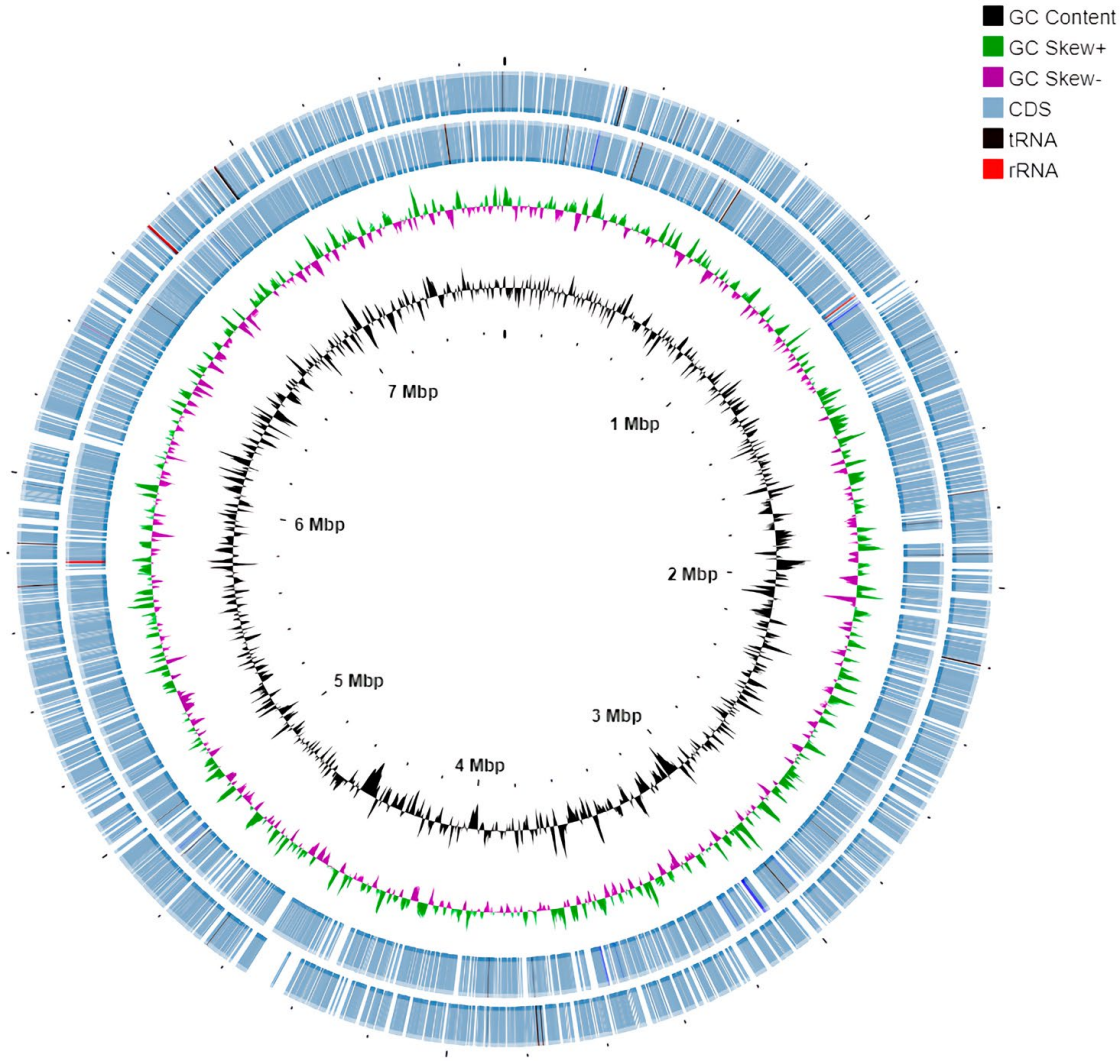
Circular assembled genome of *Capilliphycus salinus* ALCB114379 showing, via a color scheme, GC content and skew, coding sequences (CDS) regions, predicted regions for transfer RNA (tRNA), and ribosomal RNA (rRNA).

Long‐read genome sequencing of *Capilliphycus salinus* strain ALCB114379 generated 326,539 reads (2.9 Gb), with 322,185 reads (98.67%) classified taxonomically. The complete Cyanobacterial genome was assembled into a single circular structure (Figure 1 and Figure S1) with a total size of 7,712,904 bp, a G + C content of 43.23%, and 111.78× coverage. Automatic gene annotation predicted 6204 protein‐coding genes, seven rRNA genes, and 52 tRNA genes. The GC composition showed fluctuation, with asymmetric GC skewing throughout the genome. The quality of the assembly was confirmed with 99.67% genome completeness and a low contamination level (0.87%).

### Phylogenetic analysis

**FIGURE 2:**
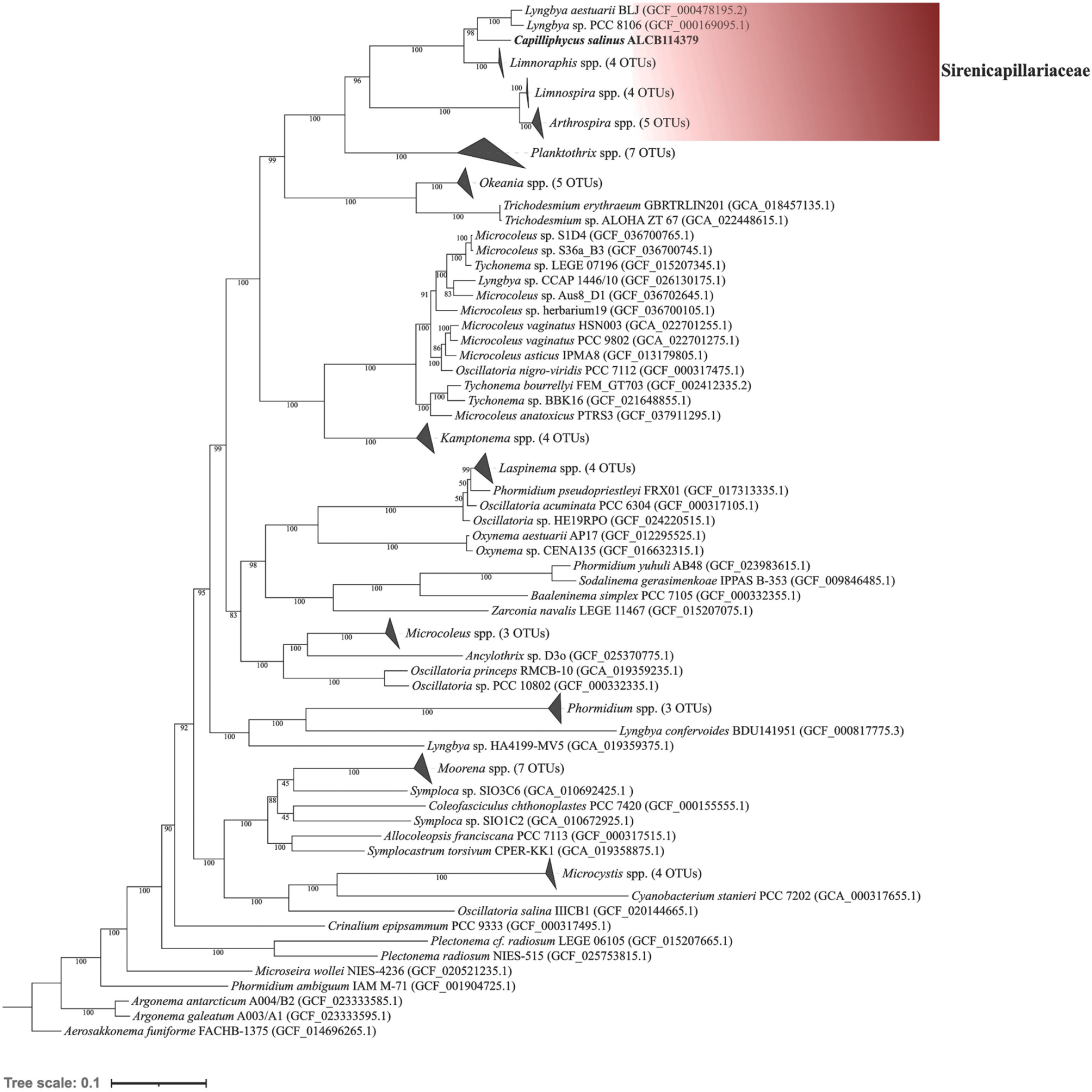
Maximum likelihood phylogenomic tree based on 120 bacterial single‐copy conserved marker proteins highlighting the relationship between the newly assembled *Capilliphycus salinus* ALCB114379 genome (in bold) with other species of Cyanobacteria retrieved from NCBI. The Sirenicapillariaceae clade is highlighted in red. Bootstrap values (>60%) are displayed at the relevant nodes.

The phylogenetic analysis revealed that *Capilliphycus salinus* ALCB114379 clustered within a monophyletic clade alongside other *Capilliphycus* strains, with 100% posterior probability, indicating strong robustness of this clade (Figure S2). The phylogenomic tree placed *C. salinus* ALCB114379 in a distinct branch, forming a clade with two genomes annotated as *Lyngbya* (*L. aestuarii* BLJ and *Lyngbya* sp. PCC8106), with high bootstrap support (98%). These two “*Lyngbya*” genomes further grouped into a subclade, with 100% bootstrap support, *C. salinus* ALCB114379 acting as an external taxon. This clade, consisting of all three strains sharing a common ancestry, was positioned as a major clade alongside a subclade of *Limnoraphis* strains, which also showed 100% bootstrap support (Figure 2).

### Identification of biosynthetic gene clusters

The predicted genes automatically annotated in the Cyanobacterial genome were categorized by BlastKOALA according to biological subsystems (Figure S3). Of the 6204 predicted protein‐coding genes, only 2197 (35.2%) were categorized, indicating significant unknown metabolic diversity. A large set of genes was related to “Protein Families Responsible for Signaling” (9.6%) and “Cellular and Genetic Information Processing” (8.87%), followed by “Energy Metabolism” (8.1%).

**FIGURE 3:**
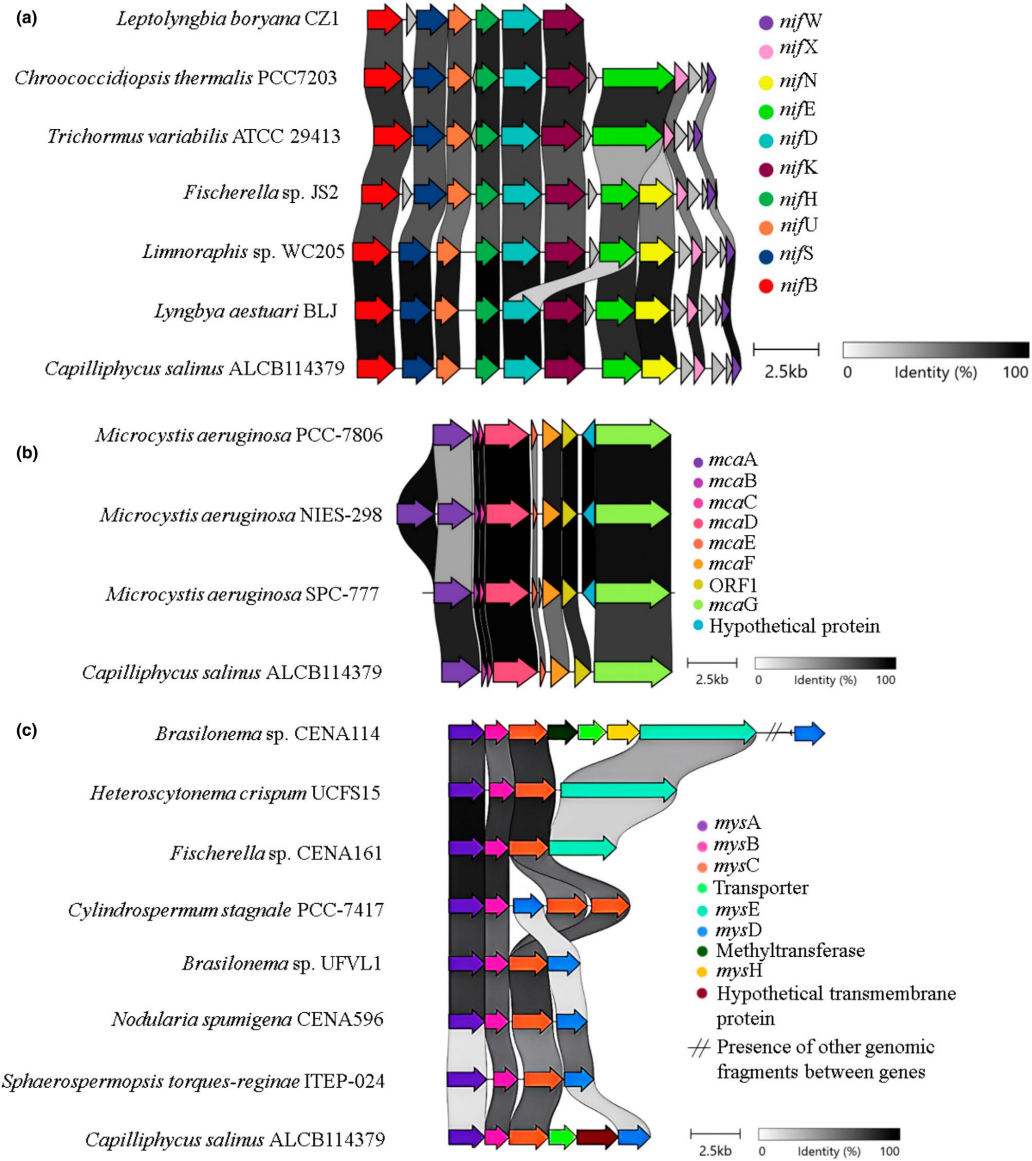
(a) Identity comparison of the biosynthetic gene cluster related to nitrogenase production (*nif*) between *Capilliphycus salinus* ALCB114379 and known strains of nitrogen‐fixing Cyanobacteria; (b) Identity comparison of the biosynthetic gene cluster related to the production of microcyclamide (*mca*) among *C. salinus* ALCB114379 and known MCA‐producing Cyanobacterial strains; (c) Identity comparison of the biosynthetic gene cluster related to the production of MAAs (*mys*) among *C. salinus* ALCB114379 and known MAA producing Cyanobacterial strains.

Interestingly, within the “Energy Metabolism” category, the BGC for the production of molybdenum‐type nitrogenase, composed of 10 genes (*nif*BSUHDKENXW), was identified. Within the category of lowest correspondence “Biosynthesis of Other Secondary Metabolites” (0.27%), the canonical cluster of four genes (*mys*ABCD) was identified, related to the biosynthesis of several MAAs, molecules with photoprotective activities. The nitrogenase (Figure 3a) and MAAs (Figure 3c) BGCs exhibited an average genetic identity of 94%, indicating strong conservation across strains, whereas the microcyclamide BGC showed an average identity of 88%, suggesting slightly more variation among strains (Figure 3b).

Using antiSMASH, a BGC associated with microcyclamide, a cytotoxic cyclic hexapeptide, composed of seven genes (*mca*ABCDEFG), was also noted. Codon‐bias analysis indicated that the microcyclamide BGC has the same frequency of occurrence as synonymous codons in DNA coding, suggesting that the BGC is well integrated into this Cyanobacterial strain genome (Figure S4).

### Chemical analysis of mycosporine‐like amino acids

Quantitative analysis of MAAs in biomass extracts of *Capilliphycus salinus* ALCB114379 detected porphyra‐334 at a low abundance (0.04 ± 0.02 μg · mg^−1^) and shinorine at a substantially higher abundance (3.04 ± 1.75 μg · mg^−1^). Chromatographic peaks consistent with palythine were observed. However, palythine was detected only at trace levels and was below the limit of quantification (LOQ < 0.005 μg · mg^−1^; Figure S5). Within the constraints of quantifiable measurements, Tukey's test did not detect a significant difference between the measured porphyra‐334 values and the trace/near‐LOQ palythine signal (Figure S6).

## DISCUSSION

### *Capilliphycus* placement in the Oscillatoriales

*Capilliphycus* was first described by Caires et al. (2018) and nomenclaturally validated by Caires (2019) with two species: *C. salinus*, isolated from hypersaline tidal pools in a supralittoral zone, and *C. tropicalis*, isolated from a protected reef region in a mediolittoral zone (Bahia State, Brazil). The first was elected as the type species for this genus and *C. salinus* ALCB114379 as the reference strain. Two additional species were described in a subsequent study: *C. flaviceps*, observed within floating marine mats of larger filamentous cyanobacteria in Florida, United States, and *C. guerandensis*, observed in hypersaline pools within inland salt flats in Guérande, Terre de Sel, France (Berthold et al., 2022). The occurrence of this genus was also reported in India (Caires et al., 2018). These studies suggest that *Capilliphycus* is largely restricted to saline environments across multiple continents.

In the present study, we provide a genome sequence for the *Capilliphycus*, specifically the complete circular genome of the reference strain *C. salinus* ALCB114379. The genome size and G + C content were comparable to those reported for *Limnoraphis robusta* CS‐951 (7,314,117 bp and 41.6% G/C) and *Limnoraphis* sp. WC205 (7,290,090 bp and 41.8% G/C), two other Oscillatoriales genomes within the Sirenicapillariaceae family (Dreher et al., 2022; Komárek et al., 2013; Willis et al., 2015). This closeness in genome size and GC content might indicate conservation of shared adaptive traits with the taxonomically related strains (Larsson et al., 2011). The asymmetric GC skew agreed with previous reports of what is most commonly found in sequenced Cyanobacterial genomes (Dextro, Delbaje, Freitas, et al., 2023; Ohbayashi et al., 2020). Both *C. salinus* ALCB114379 and *Limnoraphis* spp. share filamentous structures and the presence of aerotopes, highlighting convergent features despite their distinct ecological behaviors. Aerotopes, typically associated with buoyancy in planktonic cyanobacteria, are present in both genera. Although *Limnoraphis* spp. relies on aerotopes to navigate and remain suspended in freshwater planktic environments, the presence of aerotopes in *C. salinus* ALCB114379 is intriguing, given its epilithic lifestyle in tidal pools. The strain ALCB114379 also forms dense, fasciculate mats, in contrast to the more solitary or loosely aggregated filaments of *Limnoraphis* spp. Furthermore, *C. salinus* ALCB114379 has cylindrical cells organized into robust mats, whereas *Limnoraphis* spp. features flattened, discoid cells and sightly flexuous filaments (Caires et al., 2018; Komárek et al., 2013).

The 16S rRNA gene phylogenetic analysis in this study updates the findings of Caires et al. (2018). The resulting phylogenetic tree was consistent with recent literature (Berthold et al., 2022; Wang et al., 2023), clustering *Capilliphycus* representatives with >98% 16S rRNA gene sequence identity and placing them as sister taxa to *Limnoraphis* spp.

The phylogenomic tree for *Capilliphycus salinus* ALCB114379 was less robust than the 16S rRNA gene phylogeny tree due to the limited availability of genome sequences. Despite this, the phylogenomic analysis revealed a tree topology consistent with the 16S rRNA gene phylogeny from this study and previous research (Berthold et al., 2022; Caires et al., 2018), particularly emphasizing the close relationship between *C. salinus* ALCB114379 and *Limnoraphis* strains. The phylogenomic analysis revealed that *C. salinus* ALCB114379 clustered within a major clade alongside *Limnoraphis* and *Lyngbya*‐like strains yet was distantly related to the true *Lyngbya* clade, which includes the reference strain of this genus, *Lyngbya confervoides* BDU 141951 (Chandrababunaidu et al., 2015). A recent investigation on mangrove biofilms isolated *Capilliphycus* strains, and the phylogeny based on their 16S rRNA sequences corroborated the clustering of *Capilliphycus* and *Limnoraphis* with shared ancestry (Wang et al., 2023). The two *Lyngbya aestuarii* BLJ and *Lyngbya* sp. PCC 8106, which clustered with *C. salinus* ALCB114379 in the phylogenomic tree, were also positioned within the major *Capilliphycus* clade in the 16S rRNA gene phylogeny. This result suggests that both strains should be considered for transfer to the *Capilliphycus*. Both tree topologies are likely to become more congruent as additional *Capilliphycus* genomes become available. Nevertheless, both phylogenetic trees (either based on one gene or multiple genes) support the classification of *Capilliphycus* as a distinct genus, reinforcing its taxonomic status.

In a survey of the Cyanobacterial genome assemblies available in the NCBI (*n* = 6472 assemblies), Dextro et al. (2021) reported that assemblies assigned to Oscillatoriales accounted for 12.14% of the total. Within the Oscillatoriales order, assemblies were predominantly at the contig (32.95%) or scaffold (61.19%) level. Dextro et al. (2021) also noted that most Oscillatoriales assemblies lacked genus‐level identification (>67%) and that the best‐represented genera were *Moorena*, *Microcoleus*, and *Okeania* (each with >25 assemblies). In total, 26 genera within Oscillatoriales had genomes deposited, with most samples originating from the Northern Hemisphere, particularly the United States, Czech Republic, and China (Dextro et al., 2021). A considerable portion of assemblies (>15%) came from unknown origins, which limits their utility for metanalytic studies and highlights the importance of following proper sampling and isolation protocols to ensure the integrity of genome data (Dextro et al., 2024). The assembly of *Capilliphycus salinus* ALCB114379 is one of the few genomes from Brazil, contributing to an enhanced understanding of the evolution and behavior of underrepresented cyanobacterial strains from tropical habitats.

### Diazotrophic potential

*Capilliphycus salinus* ALCB114379 encodes the core nitrogenase gene set (*nif*BENHDK) required for assembly of a molybdenum–iron nitrogenase, which catalyzes the reduction of atmospheric N_2_ to NH_3_. These *nif* genes are broadly conserved among diazotrophs and are central to nitrogenase function (Nichio et al., 2025). In *C. salinus* ALCB114379, the *nif* cluster includes *nif*B (FeMo‐cofactor biosynthesis), *nif*E/*nif*N (FeMo‐cofactor scaffold), *nif*H (dinitrogenase reductase), and *nif*D/*nif*K (dinitrogenase; Fani et al., 2000; Jiang et al., 2022). This genomic repertoire supports nitrogen‐fixation potential, but it does not by itself demonstrate activity. Future validation could therefore focus on physiological assays under N‐deficiency conditions, using acetylene reduction assays and stable‐isotope approaches to confirm N_2_ fixation.

Synteny‐based comparative genomic analysis revealed that other cyanobacteria, such as *L. aestuarii* BLJ and *Limnoraphis* sp. WC205, also possess homologous *nif* genes essential for N_2_ fixation. Like *C. salinus* ALCB114379, these cyanobacteria carry the genetic framework required for N_2_ fixation, with conserved structural genes such as *nif*BSUHDKENXW, which are located within these strains. This genetic similarity suggests a shared evolutionary adaptation for nitrogen fixation in these cyanobacteria. In addition to the core *nif* genes, the presence of other critical genes such as *nif*S, *nif*U, *nif*X, and *nif*W further supports the nitrogen‐fixing potential of these cyanobacteria. The *nif*S gene encodes cysteine desulfurase, which provides sulfur for the formation of iron–sulfur (Fe‐S) clusters, a crucial component of the nitrogenase enzyme. The *nif*U gene encodes a scaffold protein that facilitates the assembly of Fe‐S clusters (Zhao et al., 2007). The *nif*X gene is involved in the biosynthesis of the iron‐molybdenum cofactor (FeMo‐co), and the *nif*W gene plays a protective role in maintaining nitrogenase activity under fluctuating environmental conditions, such as the presence of oxygen (Arnold et al., 1988).

The role of the *nif*W gene in maintaining nitrogenase activity, even under aerobic conditions, is particularly important. A study by Nonaka et al. (2019) demonstrated that the filamentous, non‐heterocyte‐forming Cyanobacterium *Leptolyngbya boryana* could fix nitrogen under aerobic conditions, a capability linked to the presence of the *nif*W gene. This gene ensures the continued activity of nitrogenase despite the persistent presence of oxygen, a byproduct of photosynthesis. Given that *Capilliphycus salinus* ALCB114379 shares several of these key genes, including *nif*W, it is likely that this strain also possesses the ability to fix nitrogen in oxygenic environments. Several Oscillatoriales benthic Cyanobacteria, such as *L. boryana*, *Hydrocoleum glutinosum*, *Oscillatoria bonnemaisonii*, and *Lyngbya majuscula*, have been shown to be major contributors to nitrogen fixation on coral reefs of the Caribbean basin (Brocke et al., 2018). *Capilliphycus salinus* ALCB114379 is a benthic Cyanobacterium with putative diazotrophic potential and may contribute to nitrogen inputs in supralittoral microbial mats/tidal‐pool communities where it occurs, given that cyanobacteria in hypersaline mat systems can function as atmospheric nitrogen fixers (Ramos et al., 2017). However, broader floristic coverage of marine benthic cyanobacteria along the Brazilian coast remains limited, constraining biogeographic inferences (Crispino & Sant'Anna, 2006).

### Two natural products found in *Capilliphycus salinus* ALCB114379

The BGC for microcyclamide identified in *Capilliphycus salinus* ALCB114379 exhibited collinearity with three strains of the genus *Microcystis*. Two of these strains, *M. aeruginosa* NIES298 and *M. aeruginosa* PCC 7806, possess the complete BGC required for microcyclamide biosynthesis, including the genes *mca*ABCDEFG (Ziemert et al., 2008). Two open reading frames (ORF1 and ORF2), located between the *mca*F and *mca*G genes, are also present but are not directly involved in microcyclamide synthesis. In *C. salinus* ALCB114379, the *mca*ABCDEFG genes and ORF1 are present, whereas ORF2 is absent. Several studies have identified various microcyclamide analogs, all exclusively within the *Microcystis* genus (Freitas et al., 2023; Zafrir‐Ilan & Carmeli, 2010; Ziemert et al., 2008). To our knowledge, microcyclamide has not been observed in another Cyanobacterial genus (*Capilliphycus*), raising new questions about the functional role of this molecule. This novelty is particularly intriguing, especially considering the taxonomic distance between these two Cyanobacteria, which differ at the order level and in their environmental occurrences.

Codon‐bias analysis comparing the microcyclamide BGC to the genomic background revealed a largely consistent codon‐usage profile, suggesting compositional adaptation to the host genome. Although codon usage alone cannot resolve the origin or timing of acquisition, we complemented this analysis with a genome‐wide compositional HGT screen using Alien Hunter. This scan did not flag the cyanobactin/microcyclamide locus as compositionally atypical, providing additional evidence against a recent HGT event. However, we note that older transfers can become difficult to detect. Accordingly, we interpret these results as consistent with either vertical inheritance or an ancient acquisition followed by genome‐wide compositional convergence.

Despite the widespread occurrence of microcyclamide among *Microcystis* ecotypes, the ecological function of these compounds remains unknown. A recent study evaluated the ecotoxicological activity of a microcyclamide analog and reported cardiotoxic effects in *Danio rerio* embryos (Freitas et al., 2023). Additionally, a cytotoxic effect was observed on murine carcinoma cells with two types of microcyclamides, 7806A and 7806B (Ishida et al., 2000). Although the BGC for microcyclamide was observed to be intact and integrated into the genome of *Capilliphycus salinus* ALCB114379, further investigation is required to confirm the production of this compound and to characterize this microcyclamide analog.

The collinear BGC related to MAAs observed in *Capilliphycus salinus* ALCB114379 is structurally like several other Cyanobacterial strains already known to produce a wide variety of MAAs (Dextro, Delbaje, Geraldes, et al., 2023; Geraldes et al., 2020). Recently, the organized presence of MAA‐related BGCs containing the *mys*D gene in Cyanobacterial genomes has been associated with a high probability of production, as this organized cluster is rare in the phylum. It is in only 11.8% of all genome sequences at NCBI and encodes a *mys*ABCD gene cluster arrangement (Dextro, Fiore, & Long, 2023). Strains of Oscillatoriales are underrepresented in the evaluation of MAAs. Only a few strains, such as *Lyngbya* and *Microcoleus*, have been described as producers (Karsten et al., 1998; Karsten & Garcia‐Pichel, 1996), with the organized BGC identified in only 21% of the more than 450 available Oscillatoriales genome assemblies in NCBI, each with over 90% completeness (Dextro, Fiore, & Long, 2023). Identified in mat communities and Cyanobacterial isolates from intertidal regions and mangroves, MAAs are associated with photoprotection and osmotic regulation, which facilitate survival in extreme habitats (Garcia‐Pichel & Castenholz, 1993). Therefore, strains isolated from such habitats that possess the organized *mys* gene cluster are good targets for further evaluation of MAA biosynthesis.

The MAAs quantitative analysis of *Capilliphycus salinus* ALCB114379 biomass extracts showed that out of the three MAA standards evaluated, a significant, high concentration of shinorine was detected (>3.00 μg · mg^−1^), with a small production of porphyra‐334 (<0.05 μg · mg^−1^) and trace amounts of palythine (below the detection limit). These MAAs were produced without any chemical stress or physical stimulus, such as UV exposure, representing the occurrence of a constitutive biosynthesis. Similar constitutive production yield has been described in the intertidal strain *Halotia branconii* CENA392 (Dextro et al., 2023) but for porphyra‐334 and associated with a different gene cluster configuration (*mys*ABCE). Other Cyanobacterial strains from Brazil have been described as constitutive MAA producers of shinorine, such as *Nodularia spumigena* CENA596 (0.07 ± 0.04 μg · mg^−1^), which is also a marine strain, and *Sphaerospermopsis torques‐reginae* ITEP‐024 (3.14 ± 0.98 μg · mg^−1^), a freshwater Cyanobacterium from the Northeast of Brazil (Dextro, Delbaje, Geraldes, et al., 2023). Both were cultivated at the same Z8 medium as *C. salinus* ALCB114379 in this study. The concentration value comparison with these other strains highlights the biotechnological potential of *C. salinus* as a natural source of shinorine.

The combination of the high‐performance liquid chromatography (HPLC) quantitative extract analysis, the presence of the colinear gene cluster related to MAAs biosynthesis, and the knowledge of halophytic Cyanobacteria use MAAs as osmotic solutes to survive and thrive in high salinity environments (Feng et al., 2023; Oren, 1997; Waditee‐Sirisattha et al., 2014) strengthens the overall description of *C. salinus* ALCB114379 as one of the few Oscillatoriales described in the literature to produce MAAs. These specialized metabolites serve multiple protective purposes (against UV, salinity, and dehydration) and can be associated with the fitness of an organism capable of colonizing salt tidal pools in the supralittoral zone of a beach.

## CONCLUSIONS

Through comprehensive genomic analysis and targeted profiling of MAA production, this study presented the genome of *Capilliphycus salinus* ALCB114379 and identified it as a potential diazotrophic Cyanobacterium, likely capable of production of diverse arrays of bioactive compounds. These findings contribute to the enhancement of taxonomy and the understanding of Cyanobacterial diversity, while highlighting the potential of this strain to synthesize microcyclamides and its ability to constitutively produce MAAs, two metabolite classes with notable biotechnological relevance. This underscores the remarkable capacity of Cyanobacteria to diversify and produce a wide array of bioactive compounds, revealing both their ecological plasticity and their significant biotechnological potential.

## AUTHOR CONTRIBUTIONS

**Gabriel Schimmelpfeng Passos:** Conceptualization (equal); data curation (equal); formal analysis (lead); investigation (equal); methodology (equal); software (equal); writing – original draft (equal); writing – review and editing (equal). **Rafael Barty Dextro:** Conceptualization (equal); data curation (equal); investigation (equal); methodology (equal); software (equal); writing – original draft (equal); writing – review and editing (equal). **Mauricio Junior Machado:** Conceptualization (equal); data curation (equal); software (equal); writing – review and editing (equal). **Anderson Miguel Teixeira Feitosa:** Data curation (equal); software (equal). **Núbia Pereira Silva:** Data curation (equal); software (equal). **Renata Beatriz Cruz:** Data curation (equal); investigation (supporting). **Ana Paula Dini Andreote:** Conceptualization (equal); data curation (equal); writing – review and editing (equal). **Ernani Pinto:** Methodology (equal); resources (equal); writing – review and editing (equal). **Marli Fatima Fiore:** Conceptualization (equal); funding acquisition (lead); resources (equal); supervision (lead); writing – review and editing (equal).

## Supporting information


**Figure S1.** Circular assembled genome of *Capilliphycus salinus* ALCB114379 showing, via a color scheme, GC content and skew, coding sequences (CDS) regions, predicted regions for transfer RNA (tRNA), and ribosomal RNA (rRNA). Corresponds to Figure 1 in the manuscript.
**Figure S2**. Phylogenetic analysis of *Capilliphycus salinus* ALCB114379 (in bold) based on the 16S rRNA gene and other Cyanobacteria reference strains. The *Capilliphycus* clade is highlighted in red. Bootstrap values (>60%) are displayed at the relevant nodes.
**Figure S3**. Number of predicted genes found within the assembled genome of *Capilliphycus salinus* ALCB114379 according to each biological subsystem of BlastKOALA (v2.2).
**Figure S4**. Codon fraction analysis between the *Capilliphycus salinus* ALCB114379 genome and its microcyclamide biosynthetic gene cluster (BGC). The blue dotted lines represent the tRNAs paired with their respective anticodons.
**Figure S5**. Chromatograms of three mycosporine‐like amino acids (MAAs): Shinorine, porphyra‐334, and palythine. Panels a, c, and e depict the chromatographic profiles of the corresponding MAA standards, while panels b, d, and f represent the chromatograms of biomass extracts from *Capilliphycus salinus* ALCB114379. Chemical structures of the analyzed MAAs are included for reference.
**Figure S6**. Comparison of MAA production levels in *Capilliphycus salinus* ALCB114379 as assessed by Tukey's test. Concentrations of the mycosporine‐like amino acids (MAAs) palythine and porphyra‐334 were not significantly different from each other. However, shinorine production was significantly higher compared to both palythine and porphyra‐334, with Pr > *F* = 0.0161.

## Data Availability

Data supporting the findings of this research are openly available upon request via email to the corresponding author of this article. The genome sequence has been deposited in the NCBI RefSeq database under accession GCF_047276495.1, and the LC–MS/MS raw data have been deposited in MassIVE under accession MSV000100617 (dataset DOI: 10.25345/C5SQ8QX35).
