# Supplementary material for: Genomic and secondary metabolites of the marine cyanobacterium Capilliphycus salinus ALCB114379
Source: J Phycol. 2026 May 9;62(3):904–16. doi: 10.1111/jpy.70174 (PMC13280781; doi:10.1111/jpy.70174)
Supplement: Supplementary file 1 — Figure S1. Circular assembled genome of Capilliphycus salinus ALCB114379 showing, via a color scheme, GC content and skew, coding sequences (CDS) regions, predicted regions for transfer RNA (tRNA), and ribosomal RNA (rRNA). Corresponds to Figure 1 in the manuscript. Figure S2. Phylogenetic analysis of Capilliphycus salinus ALCB114379 (in bold) based on the 16S rRNA gene and other Cyanobacteria reference strains. The Capilliphycus clade is highlighted in red. Bootstrap values (>60%) are displayed at the relevant nodes. Figure S3. Number of predicted genes found within the assembled genome of Capilliphycus salinus ALCB114379 according to each biological subsystem of BlastKOALA (v2.2). Figure S4. Codon fraction analysis between the Capilliphycus salinus ALCB114379 genome and its microcyclamide biosynthetic gene cluster (BGC). The blue dotted lines represent the tRNAs paired with their respective anticodons. Figure S5. Chromatograms of three mycosporine‐like amino acids (MAAs): Shinorine, porphyra‐334, and palythine. Panels a, c, and e depict the chromatographic profiles of the corresponding MAA standards, while panels b, d, and f represent the chromatograms of biomass extracts from Capilliphycus salinus ALCB114379. Chemical structures of the analyzed MAAs are included for reference. Figure S6. Comparison of MAA production levels in Capilliphycus salinus ALCB114379 as assessed by Tukey's test. Concentrations of the mycosporine‐like amino acids (MAAs) palythine and porphyra‐334 were not significantly different from each other. However, shinorine production was significantly higher compared to both palythine and porphyra‐334, with Pr > F = 0.0161. [file JPY-62-904-s001.docx]

**Genomic and secondary metabolites of the marine cyanobacterium *Capilliphycus salinus* ALCB114379^1^**

Gabriel Schimmelpfeng Passos^1^, Rafael Barty Dextro^1^, Mauricio Junior Machado^1^, Anderson Miguel Teixeira Feitosa^1^, Núbia Pereira Silva^1^, Renata Beatriz Cruz^1^, Ana Paula Dini Andreote^1^, Ernani Pinto^1^, Marli Fatima Fiore^1,2^

^1^University of São Paulo (USP), Center for Nuclear Energy in Agriculture (CENA), Avenida Centenário 303, 13416-000, Piracicaba, SP, Brazil

^2^ Corresponding author e-mail: fiore@cena.usp.br

**Supplementary Material**

**
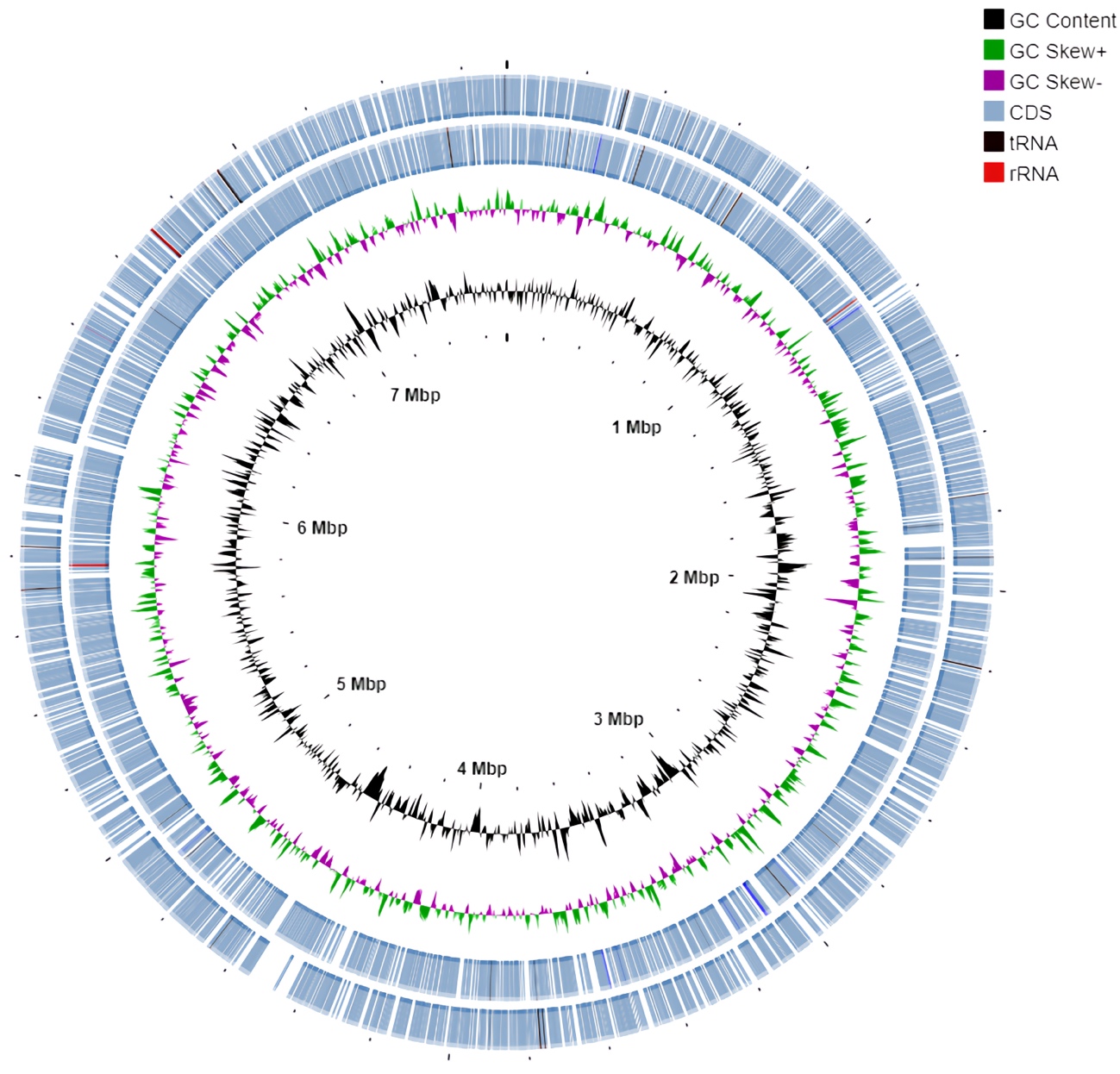
**

**Figure S1**. Circular assembled genome of *Capilliphycus salinus* ALCB114379 showing, via a color scheme, GC content and skew, coding sequences (CDS) regions, predicted regions for transfer RNA (tRNA), and ribosomal RNA (rRNA). Corresponds to Figure 1 in the manuscript.


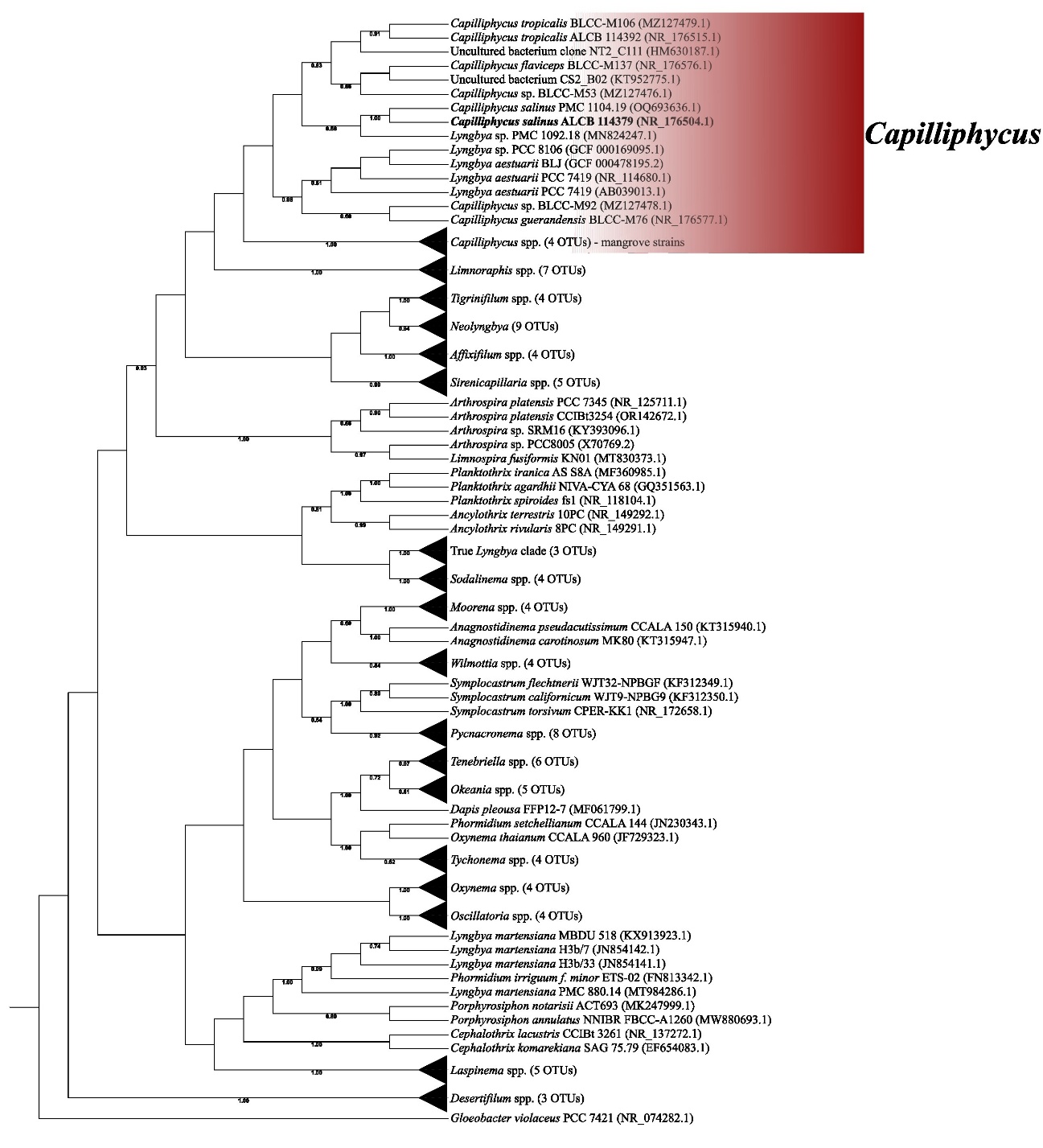


**Figure S2.** Phylogenetic analysis of *Capilliphycus salinus* ALCB114379 (in bold) based on the on 16S rRNA gene and other Cyanobacteria reference strains. The *Capilliphycus* clade is highlighted in red. Bootstrap values (>60%) are displayed at the relevant nodes.


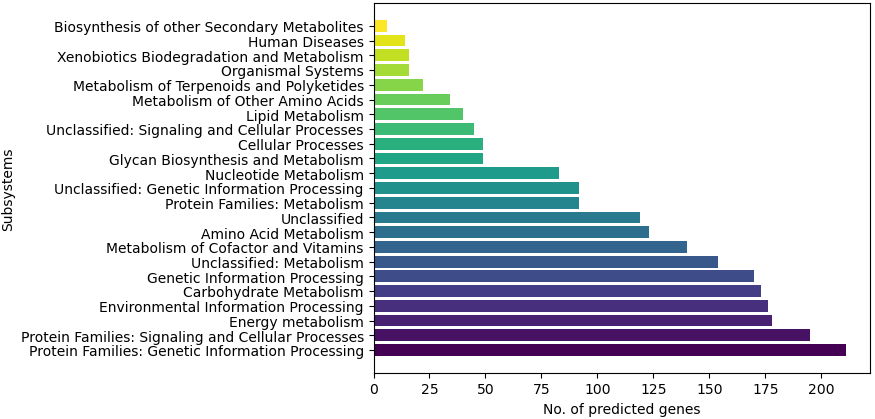


**Figure S3.** Number of predicted genes found within the assembled genome of *Capilliphycus salinus* ALCB114379 according to each biological subsystem of BlastKOALA (v2.2).


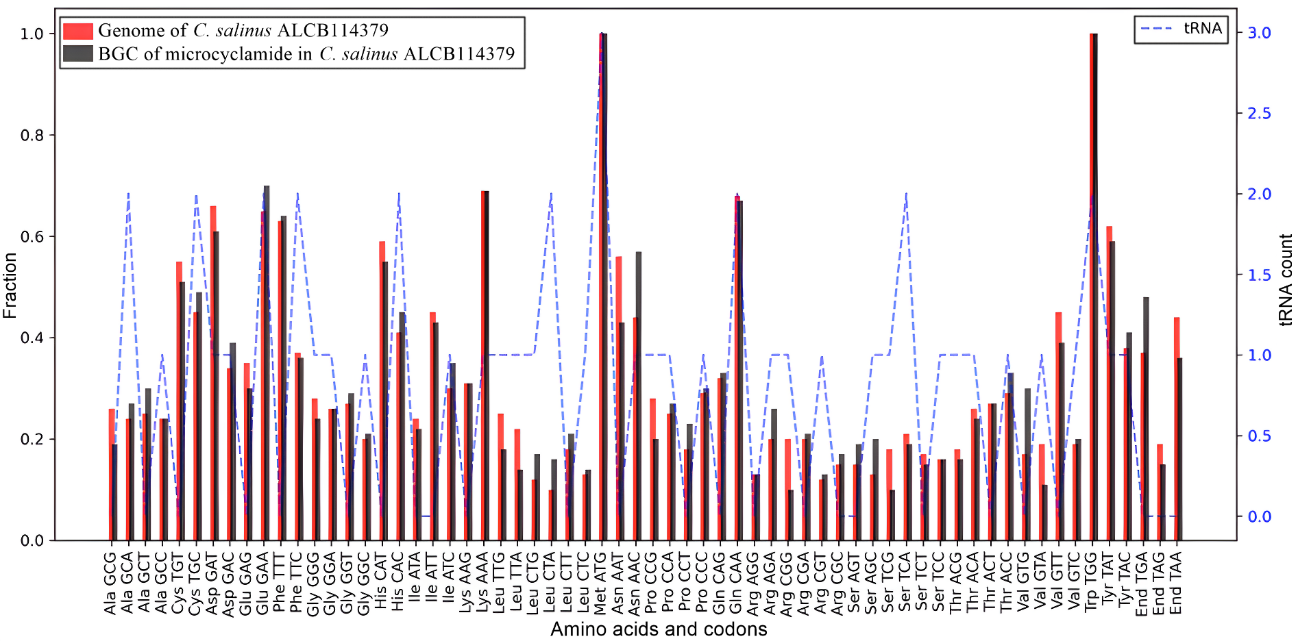


**Figure S4.** Codon fraction analysis between the *Capilliphycus salinus* ALCB114379 genome and its microcyclamide biosynthetic gene cluster (BGC). The blue dotted lines represent the tRNAs paired with their respective anticodons.


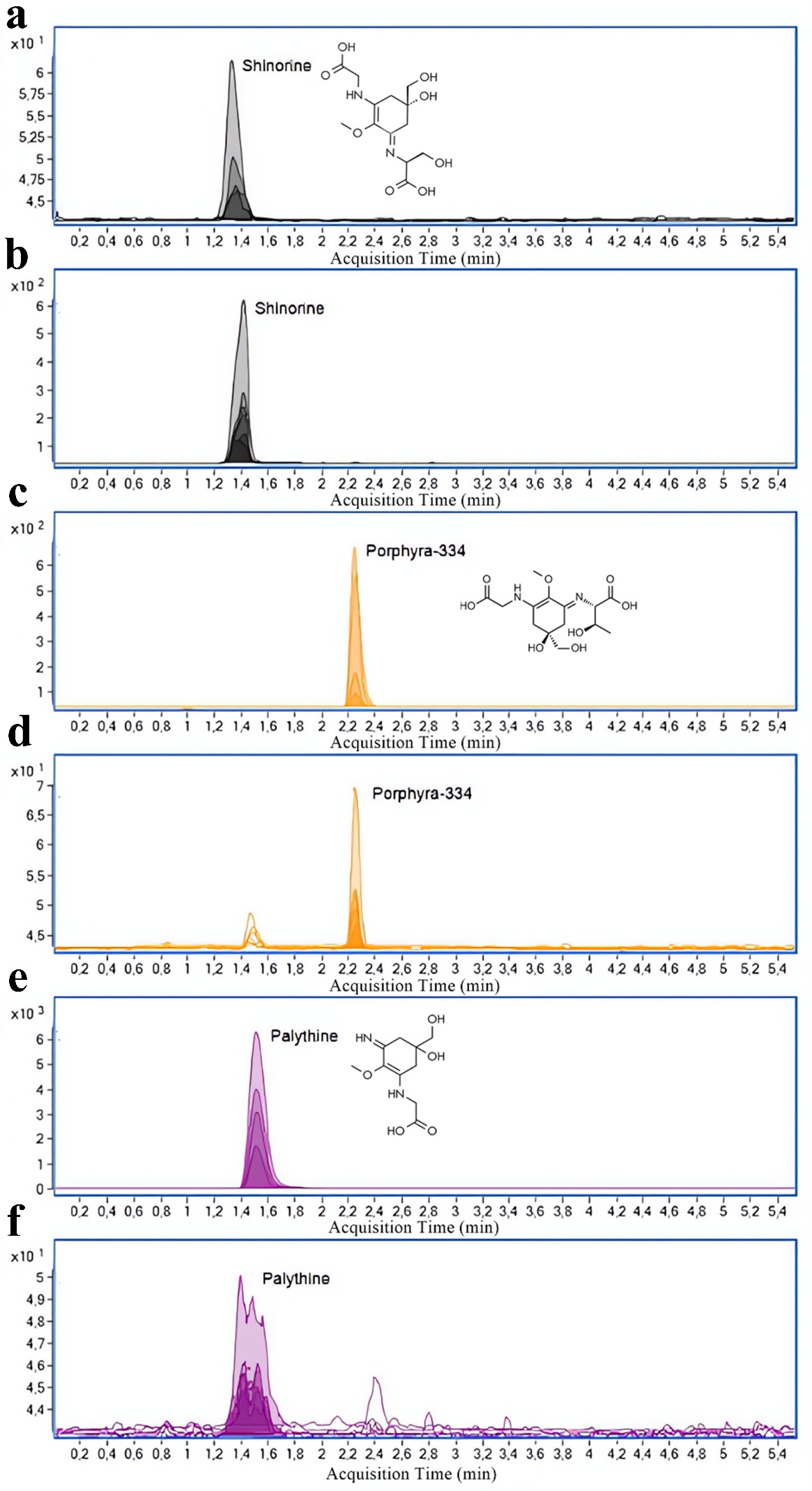


**Figure S5.** Chromatograms of three mycosporine-like amino acids (MAAs): Shinorine, porphyra-334, and palythine. Panels a, c, and e depict the chromatographic profiles of the corresponding MAA standards, while panels b, d, and f represent the chromatograms of biomass extracts from *Capilliphycus salinus* ALCB114379. Chemical structures of the analyzed MAAs are included for reference.


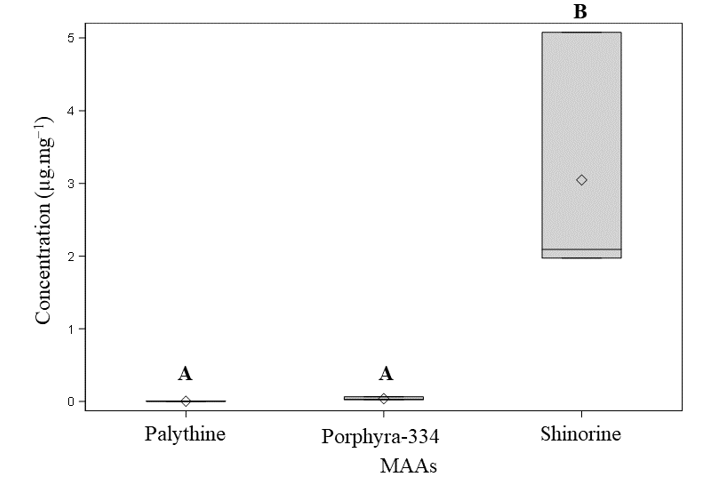


**Figure S6.** Comparison of MAA production levels in *Capilliphycus salinus* ALCB114379 as assessed by Tukey's test. Concentrations of the MAAs palythine and porphyra-334 were not significantly different from each other. However, shinorine production was significantly higher compared to both palythine and porphyra-334, with Pr > F = 0.0161.
